# From Nucleus to Membrane: A Subcellular Map of the N-Acetylation Machinery in Plants

**DOI:** 10.3390/ijms232214492

**Published:** 2022-11-21

**Authors:** Marlena Pożoga, Laura Armbruster, Markus Wirtz

**Affiliations:** Centre for Organismal Studies, Heidelberg University, Im Neuenheimer Feld 360, 69120 Heidelberg, Germany

**Keywords:** compartmentalization, co-translational modification, GNAT, N-terminal acetylation, protein turnover, PTM, stress responses

## Abstract

N-terminal acetylation (NTA) is an ancient protein modification conserved throughout all domains of life. N-terminally acetylated proteins are present in the cytosol, the nucleus, the plastids, mitochondria and the plasma membrane of plants. The frequency of NTA differs greatly between these subcellular compartments. While up to 80% of cytosolic and 20–30% of plastidic proteins are subject to NTA, NTA of mitochondrial proteins is rare. NTA alters key characteristics of proteins such as their three-dimensional structure, binding properties and lifetime. Since the majority of proteins is acetylated by five ribosome-bound N-terminal acetyltransferases (Nats) in yeast and humans, NTA was long perceived as an exclusively co-translational process in eukaryotes. The recent characterization of post-translationally acting plant Nats, which localize to the plasma membrane and the plastids, has challenged this view. Moreover, findings in humans, yeast, green algae and higher plants uncover differences in the cytosolic Nat machinery of photosynthetic and non-photosynthetic eukaryotes. These distinctive features of the plant Nat machinery might constitute adaptations to the sessile lifestyle of plants. This review sheds light on the unique role of plant N-acetyltransferases in development and stress responses as well as their evolution-driven adaptation to function in different cellular compartments.

## 1. Introduction: N-Terminal Acetylation—An Underestimated Protein Modification

**Figure 1:**
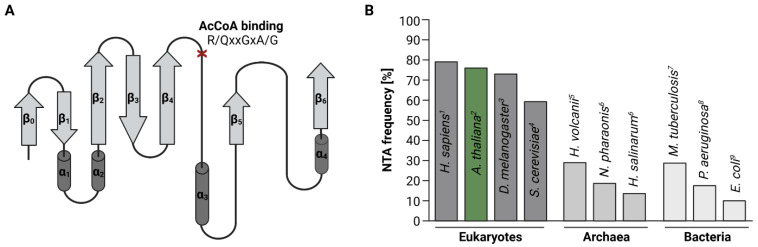
**The typical GNAT fold is conserved throughout all domains of life.** (**A**) The core GNAT fold consists of six to seven β-strands (β_0_–β_6,_ light grey) and four α-helices (α_1_–α_4_, dark grey). The loop connecting β_4_ and α_3_ contains a conserved AcCoA binding motif (R/QxxGxA/G, red cross). Differences between GNAT structures are generally confined to the N-terminal β_0_ strand. (**B**) NTA frequency in different organisms as a percentage of the whole proteome. The bars represent the estimated upper limit reported for the individual organisms (^1^: [23], ^2^: [20], ^3^: [24], ^4^: [25], ^5^: [26], ^6^: [27], ^7^: [28], ^8^: [29] and ^9^: [30]).

Protein modifications are key modulators of protein fate and are often the first-aid tool for reprogramming cells in response to developmental or environmental cues. Together with phosphorylation and ubiquitination, acetylation is one of the most pervasive protein processing events [1]. Acetylation occurs at the α-amino group of protein N-termini (N-terminal acetylation, NTA) or at the ε-amino group of internal lysine residues (lysine acetylation, KA). Both NTA and KA are present throughout all kingdoms of life and are catalyzed by N-terminal acetyltransferases (Nats) or lysine acetyltransferases (Kats) which transfer acetyl moieties from acetyl coenzyme A (AcCoA) to their respective substrates. Prokaryotic and eukaryotic Nats belong to the general control non-repressible 5 (GCN5)-related N-acetyltransferases (GNAT) superfamily which counts thousands of members in all three domains of life [2,3,4]. Despite their low overall sequence homology (3–23%), the three-dimensional fold and catalytic domains of GNATs are well conserved (Figure 1A). The core GNAT fold consists of six to seven β-strands (β_0_–β_6_) and four α-helices (α_1_–α_4_). The loop connecting β_4_ and α_3_ harbors a highly conserved R/QxxGxA/G motif, which mediates AcCoA binding [2,5,6]. In higher eukaryotes, the bulk of cytosolic proteins (>80%) is co-translationally acetylated at their N-terminus, whereas KA affects selected proteins, most prominently histones [7,8]. While KA is widely recognized as transcriptional regulator, the overall biological significance of the more prevalent NTA remains unclear [9]. At the molecular level, NTA alters the electrostatic properties of proteins by neutralizing the positive charge at their N-terminus, which results in an increased overall hydrophobicity. In addition, NTA creates a new hydrogen bond acceptor and increases the nucleophilicity and basicity of the α-amine. Taken together, these changes have profound implications for the three-dimensional structure, activity, binding properties and lifetime of individual proteins [10]. Since up to date, no N-terminal deacetylases have been identified, these changes are considered irreversible [11,12]. Hence, NTA was for a long time perceived as a nonregulated, and consequently a static, co-translational process [13]. This dogma was challenged by the identification of regulatory mechanisms for Nats and a highly diversified family of post-translational Nats in higher eukaryotes [14,15,16,17,18,19]. Specifically, in plants, the characterization of plastid-localized GNAT proteins with dual Nat and Kat activity and the phytohormone-triggered regulation of the ribosome-tethered NatA contributed to this paradigm shift [20,21,22].

This review summarizes the current knowledge on plant N-terminal acetyltransferases and their adaptation to function in different cellular compartments. Since plastids originated from prokaryotes, their NTA machinery is discussed first. Next, we focus on the eukaryotic NTA machinery, and highlight differences between photosynthetic and non-photosynthetic organisms.

## 2. The Prokaryotic Nat Machinery

While in humans and plants more than 80% of cytosolic proteins are N-terminally acetylated [20,23], the frequency of NTA declines in single-celled organisms (Figure 1B). In yeast for instance, only 60% of the proteome is N-terminally acetylated [15].

In bacteria, NTA is an even rarer event. Unlike eukaryotes, bacteria initiate protein biosynthesis with formylated methionine (fMet). Before NTA can occur, the N-terminal formyl group has to be removed co-translationally by peptide deformylase (PDF). For the majority (60%) of proteins, deformylation is followed by the excision of the initiator methionine (iMet) by methionine aminopeptidase (MetAP). Acetylation marks were found on both N-termini with and without iMet and are added by one of the three known bacterial acetyltransferases “Ribosomal modification I” (RimI), RimJ, and RimL [30,31]. Of these three enzymes, RimJ seems to be the most promiscuous since the number of N-terminally acetylated proteins in *E. coli* drops significantly upon depletion of RimJ, but not RimI or RimL. RimJ predominantly targets N-termini starting with Ser and Thr, but also Ala [32]. Despite their role as ribosome-assembly factors, Rims are absent from mature ribosomes, suggesting that their catalytic activity is purely post-translational [33].

Initially, only five endogenous proteins were reported to be N-terminally acetylated in Escherichia coli, including the ribosomal proteins S5, L7/L12, and S18 as well as the elongation factor EF-Tu and the chaperone SecB [26,34,35,36,37,38]. Recent mass spectrometry-based proteome-studies expanded this originally short list of N-terminally-acetylated proteins in *E. coli* to over 100 entries, accounting for 10% of the *E. coli* proteins with experimentally assessed acetylation status [30,32]. In *Pseudomonas aeruginosa* PA14 and *Mycobacterium tuberculosis* for instance, between 18 and 29% of the proteome were found to be N-terminally-acetylated (Figure 1B) [28,29].

Acetylation levels are similar in archaea, where 13–29% of all proteins are affected by NTA [26,27,39]. Archaea express a single conserved Nat, which exhibits a broad substrate specificity. The active site of this Nat is a hybrid of known eukaryotic Nat active sites [40,41], suggesting that the cytosolic Nats in eukaryotes derived from this ancestral form [42]. The function of NTA in archaea has only been demonstrated for individual proteins. In the salt-loving archaea Haloferax volcanii for instance, the NTA of the α1 proteasome subunit mediates the efficiency of proteolysis by altering the conformation of the channel leading up to the proteasomal core [43]. On the organismal level, the importance of NTA in archaea remains to be elucidated.

## 3. The Eukaryotic Nat Machinery

**Figure 2:**
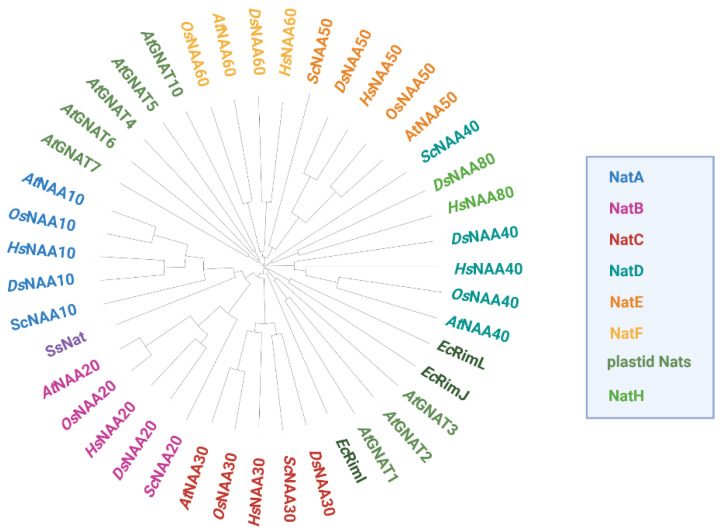
**Phylogenetic tree of Nats from different domains of life based on protein sequence comparison.** Homologous Nat sequences from the photosynthetic eukaryotes *Arabidopsis thaliana* (At) and *Oryza sativa* (*Os*), the non-photosynthetic eukaryotes *Homo sapiens* (*Hs*), *Drosophila melanogaster* (*Ds*) and *Saccharomyces cerevisiae* (*Sc*), as well as the bacterium *Escherichia coli* (*Ec*) and the archaeon *Saccharolobus solfataricus* (*Ss*) were aligned with ClustalW. For *Os*NAA50 and *Os*NAA60, only one protein could be identified by blasting the respective human orthologs against the rice proteome. The resulting phylogenetic tree was circularized with the iTOL tool (https://itol.embl.de, accessed on 20 October 2022).

So far, six evolutionary conserved Nats (NatA-F) have been identified in metazoans (Figure 2). The existence of five of those (NatA-C and NatE-F) has been experimentally confirmed in the model plant *A. thaliana* [20,44,45,46,47,48]. NatD has been proposed to exist in Arabidopsis based on the substantial homology to its human orthologue [7]. Unlike NatD and NatF, most cytosolic Nats are composed of one catalytic and one or more auxiliary subunits facilitating ribosome association and catalytic properties [49]. While NatA–E are thought to be ribosome-bound in humans and plants, NatF localizes to the plasma membrane in plants and the Golgi-membrane in humans [14,46]. In addition, a family of plastid-localized Nats (GNAT1-7 and GNAT10) with dual Kat/Nat activity was recently characterized in *A. thaliana* [21,22].

Nats are present in all plant organs (Supplemental Figure S1). While NatA–E and the plastidic Nats are widely expressed in aereal organs except for the male reproductive parts, NatF is most strongly expressed in anther and pollen. Although the distribution of the plastidic Nats among different tissues is similar, there are differences between the transcription patterns of the individual enyzmes, indicating that they might fullfil different roles in specific organs. However, in specific organs, transcript levels of Nats barely change upon various biotic and abiotic stresses (Supplemental Figure S2).

**Figure 3:**
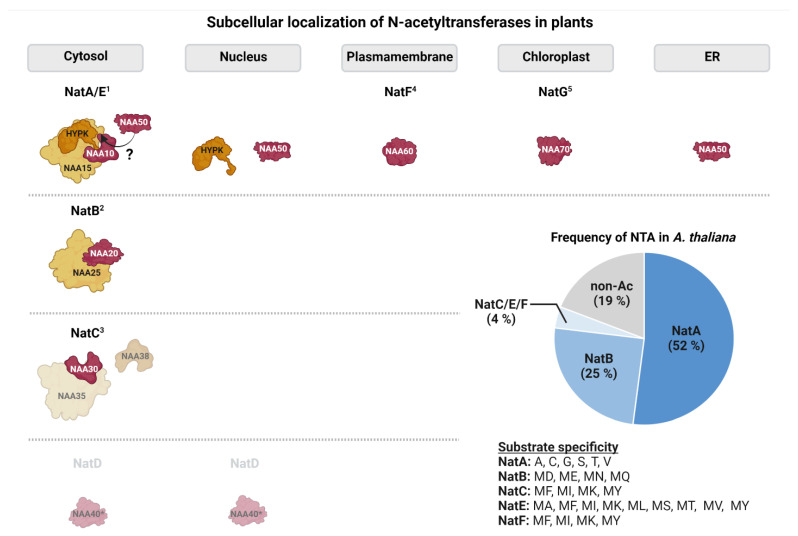
**Subcellular localization and substrate specificity of N-acetyltransferases in the model plant *Arabidopsis thaliana***. Catalytic subunits are schematically represented in red, whereas auxiliary subunits are depicted in orange. Subunits for which only predictions of subcellular localization are available are shown in lighter colors. From the plastid Nat family only NatG is shown for simplicity (^1^: [20,44,50,51,52]; ^2^: [47]; ^3^: [45]; ^4^: [46]; ^5^: [21,22], ?: debated in Arabidopsis). The pie chart shows the relative contribution of the individual acetyltransferases to the plant acetylome. Estimates are based on experimental data where acetyltransferases were assigned to acetylated N-termini based on their substrate specificity [20,53].

Furthermore, Nats may gain defined functions due to their specific subcellular compartments, which is summarized in Figure 3. In the following, we discuss the function of the Nat machinery with respect to their localization.

**Figure 4:**
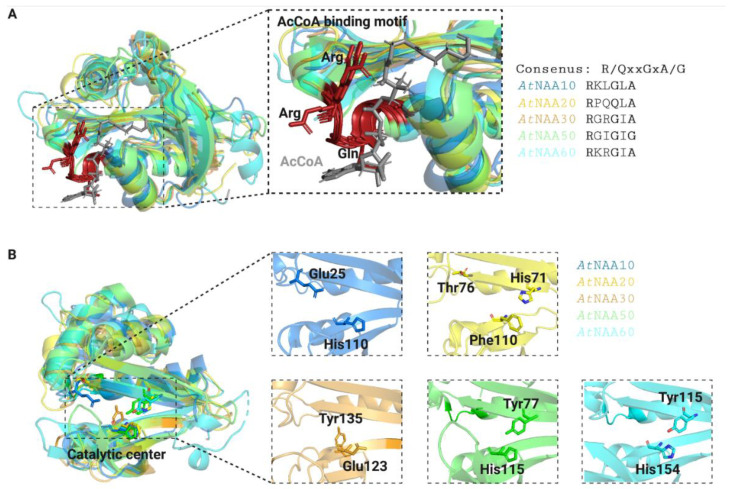
**Three-dimensional models of *Arabidopsis thaliana* Nats.** The AcCoA-binding motives (**A**) of Arabidopsis Nats are strongly conserved (shown in red with conserved residues highlighted in ribbon mode). AcetylCoA is represented in grey. The Nat catalytic sites (**B**) have distinct surface characterizations in shape, size, and electrostatic properties, which is consistent with their ability to acetylate distinct substrate pools. Catalytically important residues were either reported in [1,2] for *At*NatE and *At*NatF or estimated based on their human and yeast counterparts for *At*NatA–C [3] and are represented in stick mode. The crystal structures of *At*NAA50 (6YZZ, green) and *At*NAA60 (6TGX, cyan) were downloaded from the Protein Data Bank (https://www.rcsb.org, accessed on the 9 November 2022), whereas the three-dimensional structures of the other Nats were generated with SwissModel (https://swissmodel.expasy.org, accessed on the 9 November 2022) based on their human or yeast counterparts using the templates 6c9m.2.B (*At*NAA10, blue), 7stx.1.A (*At*NAA20, yellow) and 7l1k.1.A (*At*NAA30, orange).

The substrate specificity of Nats is largely determined by the first two amino acids of their substrate proteins [11]. Consistent with the ability of Nats to acetylate distinct N-termini, the Nat catalytic sites differ in shape, size, and electrostatic properties (Figure 4). The catalytic mechanisms of *At*NAA50 and *At*NAA60 are very similar and rely on tyrosine and histidine residues that coordinate a catalytic water molecule [46,54]. Even though the catalytic mechanisms of *At*NatA–NatC have not been uncovered yet, the residues required for catalysis in their human counterparts are conserved in plants [10].

Interestingly, some proteins are not acetylated even though based on their primary sequence they fit the recognition potential of Nats. A search in the NterDB database (https://nterdb.i2bc.paris-saclay.fr/) reveals that of 1327 nuclear-encoded putative Arabidopsis NatA substrates 179 (14%) are not acetylated. Hence, substrate recognition might depend on so far unknown determinants. Those might include the three-dimensional properties of the nascent chain or competition of Nats with other ribosome-associated factors attracted by those nascent chains.

### 3.1. The Crowded Ribosome Exit Tunnel: Ribosome-Bound Nats

Even though post-translational NTA occurs in different organelles, a substantial part of plant proteins is initially acetylated at the cytosolic ribosome [20]. Ribosomes function as protein biosynthesis machines and assist the co-translational modification, folding and translocation of newly synthesized proteins. Several of these processes occur simultaneously and require the participation of enzymes, chaperones and targeting factors that exploit ribosomes as landing pads to gain access to nascent polypeptides [55]. Hence, the association of the co-translationally operating Nats (NatA–E) with the ribosome-nascent chain (RNC) complex must be concerted spatiotemporally with other RNC-interacting factors. As the translation speed ranges between three to six polymerized amino acids per second in eukaryotes, this concerted action requires the dynamic rearrangement of protein complexes around the exit tunnel. Recent findings suggest that several ribosome expansion segments participate in the positioning of Nats above the ribosome exit tunnel, which safeguards their catalytic function [56]. Our knowledge of the interactions between Nats and other RNC-interacting factors is sparse and originates mostly from experiments conducted in metazoa. However, these findings potentially also apply to plants due to the substantial evolutionary conservation of the eukaryotic ribosome and its associated Nat machinery. Therefore, this review will also refer to the human or yeast Nat machinery for comparison.

### 3.2. NatA—The Major Eukaryotic Acetyltransferase

NatA was first discovered in *S. cerevisiae* as a heterodimeric complex comprised of the catalytic subunit NAA10 (Ard1p) and the auxiliary subunit NAA15 (Nat1p). The ribosome-association of the core NatA complex and its substrate specificity are conserved among eukaryotes [57,58,59,60,61]. However, species-specific adaptations of the interaction sites between NAA10 and NAA15 occurred as yeast NatA deletion strains can be complemented by expression of ScNatA or HsNatA but not by heterologous combinations of *Sc*NAA10 and *Hs*NAA15 and vice versa [23].

In yeast, electrostatic forces between positively charged regions on NAA15 and a negatively charged patch on the ribosomal protein L23 tether NAA15 to the ribosome and orient NAA10 towards the exit tunnel [58]. In addition to its ribosome-tethering function, *Sc*NAA15 wraps around *Sc*NAA10 in a ring-like manner and remodels the enzyme’s catalytic site. This allosteric reconfiguration induces a shift in *Sc*NAA10 substrate specificity [62]. While the NAA10 monomer post-translationally targets the α-amino groups of proteins with acidic side chains, NatA co-translationally acetylates small amino acids (Ser, Gly, Ala, Thr and Cys). Acetylation via NatA requires prior removal of the iMet by MetAP. Cryo-electron microscopy studies suggest simultaneous binding of NatA and MetAP to the RNC, allowing MetAP to hand over the processed nascent chain to NAA10 for acetylation [56,63].

In total, NatA is responsible for modifying 50% of the plant and 40% of the human proteome [23,25,53]. In agreement with its promiscuity, the depletion of NatA causes severe defects in all eukaryotes. Many studies link the loss of NAA10 activity to neurodegenerative disorders and developmental impairments (reviewed in Dörfel et al., 2015). Not only NatA deficiency, but also the presence of excess NatA has harmful effects on cells. The overexpression of NatA is linked to various types of tumour diseases, such as breast, colon, liver, lung, and prostate cancer [64,65,66,67]. A knockdown of any of the two NatA subunits leads to cell cycle arrest and apoptosis in HeLa cells, underscoring the importance of proper NatA regulation [68,69,70].

### 3.3. Global Proteome Stability Is Controlled by NatA in Humans and Plants, but Not Yeast

**Figure 5:**
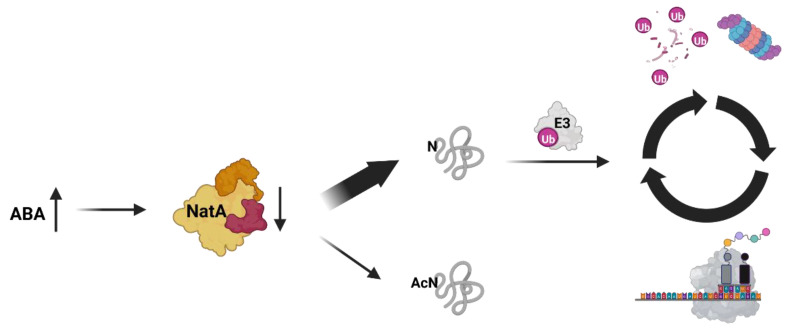
**Hypothesis of phytohormone-regulated proteome destabilization via depletion of NatA in *A. thaliana*.** In the presence of abscisic acid, NatA is depleted [20]. In consequence, less proteins are N-terminally acetylated. NatA substrates with free N-termini are recognized by up-to-date unknown E3 ubiquitin ligases, which target these proteins for degradation via the proteasome. Altogether, the depletion of NatA activity results in an increased overall protein turnover, as the degraded NatA substrates are replaced by newly synthesized proteins.

A recent study uncovered a possible molecular mechanism for the above-described inhibitory effect of NatA on apoptosis. In humans, E3 ligases named ‘inhibitor of apoptosis proteins’ (IAPs) sequester caspases and thereby impede the premature assembly of pro-apoptotic complexes. To interact with caspases, IAPs recognize IAP binding motifs (IBMs) present at the N-terminus of mitochondrial proteins. Since mitochondria have no NTA machinery, one hallmark of the IBMs is an unmodified N-terminus. Remarkably, numerous cytosolic NatA substrates harbour IBM-like sequences at their N-termini, which are masked by NTA. Upon NatA depletion, these cryptic IBMs are activated and generate a multitude of efficient IAP binders. These displace the caspases from IAPs, ultimately triggering apoptosis [71]. Whether this NatA-triggered safeguarding mechanism is conserved in plants is still uncertain. Firstly, it is currently unclear whether apoptosis exists in plants [72]. Secondly, the only IAP-like protein in Arabidopsis (AT4G19700) lacks the domain crucial for recognizing non-acetylated N-termini [73]. Nevertheless, there is evidence for the existence of non-AcN degrons in plants. Linster et al. (2022) report that in NatA-depleted mutants, proteins are degraded via the ubiquitin–proteasome system (UPS) at four times the rate observed in wildtype plants (Figure 5). This increased degradation mainly affects non-acetylated NatA substrates and is compensated by a concomitant increase in protein biosynthesis, orchestrated by the target of rapamycin, suggesting the existence of a feedback mechanism [74]. In contrast to the protective role of NatA-facilitated NTA in higher eukaryotes, the depletion of NatA has minimal effects on protein stability in yeast, suggesting that there is no widespread role for NTA in the regulation of protein turnover in this single-celled microorganism [75,76]. However, individual proteins are known to be degraded in response to their NTA status also in yeast [77].

### 3.4. NatA Is Indispensable for Plants and Regulates Biotic and Abiotic Stress Responses

Several independent studies confirmed the essentiality of both NatA subunits in *Arabidopsis thaliana* and revealed that *naa10-1* (AT5G13780) and *naa15-1* (AT1G80410) T-DNA knock-in mutants arrest development at the dermatogen to the early globular stage [20,50,51,78]. This defect can be attributed to an abnormal distribution of the growth-regulatory phytohormone auxin. In NatA mutants, no quiescent center progenitor cells are generated, underscoring the importance of NatA for early embryonic patterning [50].

**Figure 6:**
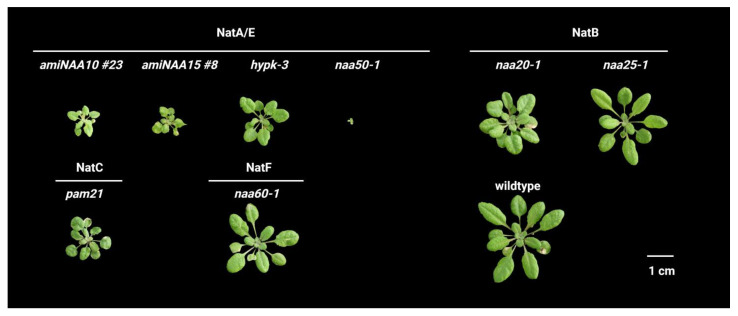
**Phenotype of *A. thaliana* mutants impaired in different components of the NTA machinery.** Mutants impaired in Nat machinery components (amiNAA10 #23, amiNAA15 #8, hypk-3 = SALK_080671, naa20-1 = SALK_027687, naa25-1 = GK-819A05, pam21 (photosyntheis affected mutant21 = SALK_119000, naa60-1 = SALK_016406C, naa70 = SALK_072318) were grown on soil for four weeks under short-day conditions, except for the *naa50* mutant (SAIL_1210_A02) which was germinated on ½ MS medium and transferred to soil after four weeks of growth. Photographs were taken by the authors and represent phenotypes of mutants previously described in independent studies cited in the main body text.

NatA knockdown mutants generated with an artificial microRNA approach still display a significant reduction of growth (Figure 6). Despite the growth retardation, the transgenic lines are fertile and remarkably drought-tolerant. This tolerance might be partially explained by the increased root-to-shoot ratio and permanently decreased stomata aperture of the NatA depleted mutants. Both traits are controlled by the drought stress-related phytohormone abscisic acid (ABA). Interestingly, endogenous ABA levels are not elevated in the NatA mutants, suggesting that the plants mimic the drought stress response independent of ABA biosynthesis. In wildtype plants, exogenous ABA administration decreases the transcription and abundance of both NatA subunits, providing the first evidence for hormonal control of NTA [20]. The enhanced turnover of non-acetylated NatA substrates upon ABA exposure might constitute an adaptation to drought as it allows for the efficient removal of stress damaged proteins (Figure 5). While the molecular connection between NatA and drought tolerance remains unclear in NatA depleted Arabidopsis mutants, differential degradation of acetylated and non-acetylated proteoforms upon desiccation has been demonstrated for the ϵ-subunit of the plastid-localized ATP synthase in wild watermelons [79].

Similarly, NatA-mediated NTA controls the stability of Nod-like receptors (NLRs) involved in plant immunity. The NatA-depleted mutant muse6 accumulates both RPM1 (AT3G07040) and SNC1 (AT4G16890) and was identified in a forward genetic screen for negative regulators of NLR-mediated autoimmunity [80,81]. In line with this finding, muse6 displays an increased resistance towards the bacterium *Pseudomonas syringae* and the virulent oomycete *Hyaloperonospora arabidopsidis*. While RPM1 is a typical NatA substrate, alternative translation initiation generates two distinct SNC1 isoforms: a non-canonical NatA substrate (Met–Met–Asp) and a NatB substrate (Met–Asp). Remarkably, acetylation via NatA or NatB impacts SNC1 stability antagonistically. Whereas acetylation by NatB stabilizes SNC1, acetylation via NatA creates an Ac/N-degron that destabilizes the immune receptor [81]. These findings suggest that environmental stimuli control protein abundance via differential NTA of specific proteoforms.

### 3.5. HYPK—A Species-Specific Modifier of NatA Activity

In most eukaryotes, the core NatA complex interacts with the auxiliary subunit HYPK [6,52,82,83]. Curiously, HYPK (Huntington Yeast Two-Hybrid Protein K) is absent in baker’s yeast [82,84]. HYPK is an intrinsically unstructured Huntingtin (HTT)-interacting protein with chaperone-like activities [85]. Mutated, aggregation-prone HTT is the cause of the incurable neurodegenerative Huntington’s disease [86]. HYPK prevents the aggregation of HTT and isolates toxic HTT aggregates in sequestration complexes [87,88]. In human cell lines, the knockdown of HYPK results in reduced cell growth, cell cycle arrest and induction of apoptosis [82,89]. These defects resemble the phenotypes observed after the knockdown of the NatA core subunits, indicating that the presence of HYPK might be required for proper NatA function. Indeed, the canonical NatA substrate PCNP is acetylated less frequently upon HYPK depletion in vivo [82]. Surprisingly, HYPK inhibits NatA activity against several substrates in vitro, which is consistent with blocking of the NatA active site by the N-terminus of HYPK in NatA/HYPK crystals from different organisms [19,83,90].

In *Arabidopsis thaliana*, the knockout of HYPK (AT3G06610) results in reduced growth as well as delayed bolting and flowering, thereby closely recapitulating the phenotype of the core NatA-depleted mutants (Figure 6). Plant HYPK also interacts with NAA10 and NAA15 and facilitates NatA activity in vivo. Hence, the loss of HYPK decreases NatA-mediated NTA, causing substantially faster turnover of NatA substrates carrying a nonAc/N-degron in Arabidopsis [91]. Similarly, in rice, the knockout of HYPK results in lowered NTA of diverse NatA substrates and induces protein translation and degradation [52]. These studies demonstrate that HYPK promotes NatA activity *in planta* and strongly suggest that HYPK modulates proteome stability by facilitating NatA activity at the ribosome. Moreover, HYPK was shown to act as an autophagy receptor in tobacco plants and as such is involved in the clearance of protein aggregates formed during proteotoxic stress [92,93]. Whether Arabidopsis HYPK also regulates autophagy independently of its role as a NatA modifier remains an open question.

### 3.6. NAA50—NatA Regulator or Independent Acetyltransferase?

The human core NatA complex can bind a second catalytic subunit termed NAA50 and thereby form the ternary NatA/E complex [19,69]. Unlike HYPK, NAA50 is present among all eukaryotes [6]. In humans, HYPK and NAA50 can bind simultaneously to the core NatA complex [10], but interaction of NatA with one of the two subunits weakens the affinity to the other [10,19]. The majority of human NAA50 is available as free monomers, whereas, in yeast, NAA50 localizes exclusively to the ribosome-bound NatA complex [59,94]. In the filamentous fungus *Chaetomium thermophilum,* NAA50 does not interact with NatA/HYPK due to its extended C-terminus which enables it to bind to the ribosome independent of NatA [95]. These species-specific differences might be explained by the diverging roles of NAA50 and the absence of HYPK in yeast. While human NAA50 is an active acetyltransferase, yeast NAA50 is catalytically dead and thought to serve as a scaffolding protein, which positions the core NatA complex above the ribosome exit tunnel [54,56,96].

The different roles of human and yeast NAA50 are also reflected in the phenotypes of knockout mutants. While the loss of *Sc*NAA50 leads to no particular phenotype except for the decreased acetylation of six NatA substrates [96], human cell lines exhibit impaired sister chromatid cohesion and chromosome condensation in response to *Hs*NAA50 depletion [94,97]. In *Arabidopsis thaliana*, the knockout of NAA50 (AT5G11340) results in severe dwarfism and infertility. Unlike *naa10* or *naa15* mutants, which fail to pass embryogenesis, *naa50* mutants develop like the wildtype until the formation of the first few leaves (Figure 6). From this time point on, *naa50* is severely growth retarded and displays premature leaf senescence, defective root cell patterning and infertility [44,50,98,99].

Like human NAA50, *At*NAA50 displays a rather broad substrate specificity covering N-termini starting with Met–Ser, Met–Thr, Met–Ala, Met–Val, Met–Leu, Met–Ile, Met–Phe, Met–Tyr and Met–Lys [44,54,90,100]. Despite this promiscuity, the enzyme is estimated to acetylate less than 4% of the plant proteome [53]. This might be due to the potential competition between NAA50 with MetAP, which usually removes the iMet of many in vitro NAA50 substrates rendering them susceptible to NTA by NatA in vivo [101]. Up to now, no in vivo substrates of *At*NAA50 have been identified [44]. However, loss of NAA50 function results in substantial growth retardation (Figure 6, and [44,99]).

The failure to identify NAA50 substrates in plants led to the hypothesis that *At*NAA50 might serve as a NatA regulator as previously shown for *At*HYPK [91]. Indeed, in humans, *Hs*NAA50 impacts the activity of *Hs*NAA10 within the NatA/E complex in vitro [19]. However, up to date, it is unclear whether NAA50 associates with the core NatA complex in Arabidopsis [44,99]. Contrary to observations made in yeast, a knockout of *At*NAA50 does not induce significant shifts in the acetylation yields of NatA substrates [44,96]. Moreover, the fact that the enzymatically active *Hs*NAA50 but not the catalytically dead *Sc*NAA50 rescues the dwarfism of *naa50* mutants hints toward a vital role of NAA50 activity in plants [44].

On the molecular level, *naa50* mutants suffer an accumulation of proteins involved in plant immunity as well as a constitutively activated ER-stress response [44,99]. In line with the upregulation of salicylic acid and ethylene signaling in the mutants, Neubauer and Innes (2020) report that NAA50 interacts with the ER-localized kinase ENHANCED DISEASE RESISTANCE1 (EDR1), which negatively regulates salicylic and ethylene signaling [102,103]. Taken together, these findings suggest that NAA50, possibly in liaison with EDR1, regulates the trade-off between plant development and defense signaling.

### 3.7. NatB—The Most Conserved Ribosome-Tethered Nat Complex

The NatB complex acetylates approximately 20% of the human and the plant proteome, and is composed of the catalytic subunit NAA20 and the auxiliary subunit NAA25 [48,104,105]. Unlike NatA, NatB is not required for vitality in humans [106]. Albeit, free NAA20 can acetylate N-termini in vitro, and the interaction of NAA20 with NAA25 is critical for in vivo NatB activity in humans, yeast and plants [48,107,108,109]. NatB preferentially acetylates nascent chains displaying an iMet followed by the acidic amino acids Glu and Asp, or their amides Asn and Gln at position two [48,81,108]. The substrate specificity and three-dimensional structure of the enzyme is conserved among animals [25,110].

Missense mutations in human NAA20 result in developmental delay, intellectual disability, and microencephaly [111]. In cell cultures, silencing of any of the two NatB subunits arrests growth, indicating that NatB is critical for cell cycle progression [105,112]. Since NatB overexpression has been linked to tumorigenesis, the complex is a promising drug target [113,114].

In yeast, the knockout of NAA20 (Nat3p) or NAA25 (Mdm20p) results in a variety of deleterious phenotypes, including slower growth, sensitivity to elevated temperatures or osmotic stress, reduced mating, defects in mitochondrial and vacuolar inheritance, as well as abnormal actin cable formation [107,108]. These phenotypes can at least partially be attributed to absent acetylation of actin in ScNatB mutants [108,115]. In addition, ∆ScNatB strains accumulate protein aggregates enriched for components of the cytoplasmic translation machinery. This aggregation hampers protein biosynthesis and triggers the activation of the stress-induced protein refolding machinery [116]. In agreement with the finding of aggregated proteins in NatB mutants, NatB is essential for the induction of autophagy, clearing protein aggregates in yeast [117]. Expression of *Hs*NAA20 fails to complement yeast ∆*Sc*NAA20. However, expression of both *Hs*NatB subunits partially rescues the phenotype of yeast NatB mutants, strongly suggesting differences in NatB complex assembly [115]. The differential maturation of the NAA20 N-terminus in humans and yeast might contribute to the observed differences in NatB assembly. While in humans, the NatB formation depends on iMet removal on the NAA20 N-terminus via MetAP, this is not the case in yeast [118].

In the model plant *A. thaliana*, orthologs of the NatB subunits NAA20 (AT1G03150) and NAA25 (AT5G58450) have been characterized. The so far known T-DNA insertions in genes encoding for NatB subunits do not entirely inhibit NTA of canonical NatB substrates but substantially decrease it [48]. These lowered NatB acetylation levels cause indistinguishable defects in embryo development and vegetative growth in the *naa20* and *naa25* mutants (Figure 6, [47,48]). Remarkably, *Hs*NAA20 but not *Sc*NAA20 rescues the *naa20* phenotype, suggesting substantial conservation of NatB complex formation between plants and humans [48]. Based on these results, the formation of a heterodimeric complex is critical for NTA of canonical NatB substrates *in planta* [7,47,48]. Since the so far available *naa20* and *naa25* mutants still display low levels of NTA on NatB substrates, it is controversially discussed whether NatB is essential in Arabidopsis [47,48].

The function of AtNatB is predominantly linked to the regulation of immunity [48,81]. Acetylation via NatB stabilizes the immune receptor SNC1 as well as the immune-activating protein SIB1 [81,119]. In line with these findings, the depletion of AtNatB results in a general downregulation of defence-related processes on the transcript level and a decreased resistance against oomycetes [48,81]. In addition, NatB mediates the stabilization of various aminocyclopropane-1-carboxylate oxidases catalyzing the rate-limiting step of ethylene biosynthesis [120].

Recently, NatB mutants were found to be hypersensitive to the reductive agent dithiothreitol as a result of a constitutive over-reduction of their cytosol [121]. In summary, NatB seems vital for stress responses in photosynthetic and non-photosynthetic organisms [48,116,121].

### 3.8. NatC—A Cytosolic Modulator of Photosynthesis

In comparison with NatA- or NatB-type N-termini, NatC-type N-termini (Met–Leu, Met–Ile, Met–Phe, Met–Trp, Met–Val, Met–Met, Met–His, or Met–Lys) are relatively rare [23,104,122,123,124,125]. The ribosome-associated NatC is composed of one catalytic (NAA30) and two auxiliary subunits (NAA35 and NAA38). Upon depletion of NAA30 in yeast, several proteins lose their defined subcellular localization to membranous compartments [126,127,128,129]. However, this is only true for individual proteins and NatC-mediated NTA has no general function in determining the subcellular localization of its substrates [130].

In human cell lines, the overexpression of *Hs*NAA30 has an anti-apoptotic effect while the depletion of NatC leads to growth arrest and cell death [123,131]. In addition, the knockdown of *Hs*NAA30 results in a decreased expression of mitochondrial proteins, a loss of mitochondrial membrane potential and mitochondrial fragmentation [132]. In yeast, all three NatC subunits are essential for the enzymatic activity of the complex [124]. Mutants depleted in any of the three complex components grow slowly on non-fermentable carbon sources, suggesting that NatC acetylates proteins involved in anaerobic energy generation [122].

In silico searches identified putative orthologs of all three NatC subunits in *A. thaliana*. While a mutation in *At*NAA30 (AT2G38130) results in dwarfism, lowered chlorophyll content and a decreased effective quantum yield of photosystem II, a T-DNA insertion in the *NAA35* gene (AT2G11000) does not yield any observable phenotypes (Figure 6). Remarkably, the ectopic expression of *At*NAA30 alone rescues yeast NatC triple mutants, suggesting that *At*NAA30 activity is not dependent on NatC complex formation. Furthermore, *At*NAA30 fails to interact with the two Arabidopsis NAA38 orthologues (AT2G23900 and AT3G11500). However, a weak interaction between *At*NAA30 and *At*NAA35 was shown in a yeast two-hybrid approach [45,122].

### 3.9. NatD—A Highly Specialized Nat Targeting Histones

The monomeric NAA40 (NatD) is a ribosome-associated, highly selective Nat. Its only known substrates are the N-termini of histones H4 and H2A. This narrow substrate specificity is conserved among yeast and humans [61,133,134,135]. In addition to acetylating histones, NAA40 displays auto-acetylation activity. In humans, NAA40 deregulation and the resulting alterations in chromatin architecture are associated with various tumor diseases, positioning NAA40 as a promising therapeutic target. In colorectal cancer cells, for instance, the downregulation of NAA40 triggers growth inhibition [136,137,138]. Although in yeast, NAA40-mediated NTA of histone H4 regulates the expression of specific genes controlling cell growth, depletion of NAA40 leads to no observable phenotype on standard culture medium except for an increased lifespan [133,139,140].

BLAST searches identified orthologues of NAA40 in red and green algae, liverworts, mosses and vascular plants, suggesting conservation of NAA40 *in plantae* [141]. While *At*NAA40 (AT1G18335) has not been biochemically characterized yet, the function of NatD seems to be conserved between humans and plants. Human NatD is tailored to recognize the Ser–Gly–Arg–Gly N-termini of its only two substrates, histones H2A and H4. These N-termini are conserved in *A. thaliana* H2A (AT1G51060) and H4 (AT2G28740), but their acetylation status was not examined up to date.

### 3.10. NatH—An Animal-Specific Actin Modifier

Recently the cytosolic NatH (NAA80) was identified in animals [18]. Contrary to NatA–E, NatH does not associate with ribosomes and acts post-translationally. The only identified substrates of the monomeric acetyltransferase are processed forms of ß- and γ-actin. NatB initially acetylates both actins. Subsequently, their N-termini are cleaved by a yet-to-be identified aminopeptidase so that NatH can acetylate the newly generated free N-termini. The maturation pathway of actins is animal specific and up to date, no NatH homolog has been identified in photosynthetic organisms [53,142].

## 4. Organellar Nats

### 4.1. Getting to the Core of the Cell: Nuclear Acetyltransferases

The significance of NTA in the nucleus is underscored by an enrichment of N-terminally acetylated proteins in the nucleus in comparison to whole cell lysates [143,144]. The catalytic subunits of all ribosome-bound human Nats (NatA–E) are found in the cytosol as well as the nucleus [14,60,82,105,135]. Even though it is unclear why Nats localize to the nucleus, there are three main hypotheses regarding the function of Nats in this particular organelle. Firstly, the enzymes might post-translationally acetylate nuclear proteins in addition to their role as co-translational acetyltransferases. However, such an activity has yet to be evidenced. Secondly, the N-acetyltransferases might act as lysine–acetyltransferases in the nucleus. Observations of lysine–acetyltransferase activity for monomeric *Hs*NAA10, *Hs*NAA40 and *Hs*NAA50 support this idea [135,145,146]. For *Hs*NAA10 and *Hs*NAA50, these findings have however been called into question since crystal structures suggest that the active sites of these enzymes cannot accommodate lysine side chains [147,148]. Recent studies suggest that oligomerization or post-translational modifications of the Nats themselves (e.g., hydroxylation) could determine whether the enzymes act as Kats [16,17]. Thirdly, acetyltransferases might act as transcriptional regulators in the nucleus. *Hs*NAA10 for instance recruits the DNA methyltransferase DNMT1 to the non-methylated E-cadherin promoter and thereby contributes to the silencing of the E-cadherin gene [149].

In plants, only *At*NAA50 has been found in the nucleus so far. In analogy to *Hs*NAA10, *At*NAA50 has been speculated to moonlight as transcriptional regulator [44]. Currently, the subcellular localization of plant Nats and their potential functions in the nucleus are understudied.

### 4.2. The Highly Diverse Family of Plastid Acetyltransferases

Only 88 of more than 3000 plastid-localized proteins are encoded in the plastome of Arabidopsis. Out of those, at least ten were found to be N-terminally acetylated [22], strongly suggesting that NTA also occurs co-translationally in the plastids of higher plants. This view is supported by the identification of a plastid ribosome-associated Nat in the unicellular green algae *Chlamydomonas reinhardtii* [150]. However, the majority of plastidic proteins are imported from the cytosol, followed by cleavage of the N-terminally located transit peptide by stromal processing peptidases. Additional peptidases subsequently remove up to three residues from the N-terminus. Together, these maturation processes result in a stunning variety of proteoforms with different N-termini that may or may not be acetylated. In total, 20–30% of all plastid-localized proteins are N-terminally acetylated, including RuBisCo, components of the light-harvesting complex and the ribosome [7,151,152,153].

Most of those N-termini are found in both an acetylated and non-acetylated form [151]. This distinguishes chloroplasts from the cytosol where the acetylation yield of individual Nat substrates amounts to >80% for the majority of analyzed proteins [20,48]. The mechanisms that govern the partial acetylation of plastidic proteins remain to be investigated. It has been proposed that the acetylation yield of individual proteins might change in response to environmental factors [141]. As the cytosolic NatA complex is under the control of the phytohormone ABA [20], similar regulatory mechanism can be conceived for plastidic Nats.

The first plastidic Nat to be identified in Arabidopsis was the monomeric GNAT4, for historical reasons, often referred to as NAA70 or NatG (AT2G39000). GNAT4 preferentially acetylates N-termini starting with Met, Ala, Ser or Thr and shows strong structural similarity to *At*NAA50. Like *At*NAA50, GNAT4 displays auto-Kat activity [22]. Later in silico searches suggested the presence of nine additional organelle-targeted Nats in the Arabidopsis proteome. Indeed, seven of those candidates (GNAT1: AT1G26220, GNAT2: AT1G32070, GNAT3: AT4G19985, GNAT5: AT1G24040, GNAT6: AT2G06025, GNAT7: AT4G28030, GNAT10: AT1G72030) localize to the chloroplasts and display dual Nat/Kat activity in vitro. According to the endosymbiont theory, plastid Nats might have evolved from prokaryotic Nats. Indeed, the closely related GNAT1-3 cluster together with the *E. coli* Nats RimJ and RimL in a phylogenetic analysis. Interestingly, GNAT4-7 and GNAT10 form a separate cluster, indicating the existence of two distinct GNAT subfamilies which have previously been referred to as “NAA70” (GNAT4-7 and GNAT10) and “NAA90” (GNAT1-3) subfamilies (Figure 2) [141]. Why plants express a whole plethora of plastid-localized Nats with broad and largely overlapping substrate specificities to acetylate less than one third of their chloroplast proteins remains an open question.

One possible explanation is that NTA is not the only function of these enzymes. This view is supported by the fact that all of the plastid Nats identified so far in *Arabidopsis thaliana* also display Kat activity [21]. Unfortunately, it is difficult to disentangle the two enzymatic activities by generating exclusive Nats or Kats via mutagenesis, since both activities are mediated by a single active site. These difficulties hamper the in vivo characterization of the dual-acting enzymes. As a result of the relaxed peptide substrate-binding pocket, the plastid-localized Nats have broad and largely overlapping substrate specificities [21]. However, a knockout of GNAT2 results in a clear phenotype with defective state transitions, indicating that it is required for the dynamic reorganization of thylakoid protein complexes in fluctuating light conditions [154]. The biological relevance of the other plastid GNATs is currently unclear.

Whether NTA contributes to protein turnover in the plastids has not been conclusively verified. There is however a positive correlation between the half-life of plastidic proteins and their NTA-frequency in *Chlamydomonas reinhardtii* [7]. The machinery which might orchestrate a targeted degradation of non-acetylated plastidic proteins has not been described yet, but the CLP protease system is a potential candidate [155].

### 4.3. Membrane-Bound Acetyltransferases

As previously discussed, the frequency of NTA appears to correlate with organismal complexity. Within the clade of eukaryotes, the presence of the membrane-associated NAA60 can at least partially explain the significantly higher NTA frequency in multicellular organisms such as the fruit fly, humans and Arabidopsis when compared to unicellular yeast, which lacks NAA60 [6,15,46].

In humans, the monomeric NAA60 post-translationally acetylates N-termini starting with Met–Leu, Met–Ile, Met–Phe, Met–Tyr or Met–Lys. The enzyme localizes to the membranous compartments of the Golgi apparatus and is critical for Golgi ribbon formation [46,156,157].

Unlike its human counterpart, Arabidopsis NAA60 (AT5G16800) localizes to the plasma membrane. The membrane association of both *Hs*NAA60 and *At*NAA60 is mainly driven by type A amphipathic α-helixes at the C-terminus of the proteins [46,157]. The difference in the localization of both enzymes might be mediated by the distinct lipid makeup of the Golgi and the plasma membrane or the diverging number and amino acid composition of the amphipathic α-helices in both Nats [46]. Instead of regulating Golgi integrity, *At*NAA60 is required for the adaptation to abiotic stress, as demonstrated by the decreased germination rate of *naa60-1* on high salt medium [46]. However, under optimal growth conditions, *naa60-1* mutants display a wildtype-like phenotype (Figure 6).

*Hs*NAA50 and *Hs*NAA60 display a high structural similarity and employ similar catalytic mechanisms. Both enzymes display Kat activity, which is marginal compared to their Nat activity [46,156,158,159]. Although the in vitro substrate specificities of both enzymes overlap, the distinct phenotypes of plant *naa50-2* and *naa60-1* mutants strongly suggest that NAA60 fails to complement the absence of NAA50 and vice versa [44,98,99]. This might be a consequence of the diverging subcellular localization of the two enzymes, which gives them access to distinct substrate pools.

## 5. Concluding Remarks

**Figure 7:**
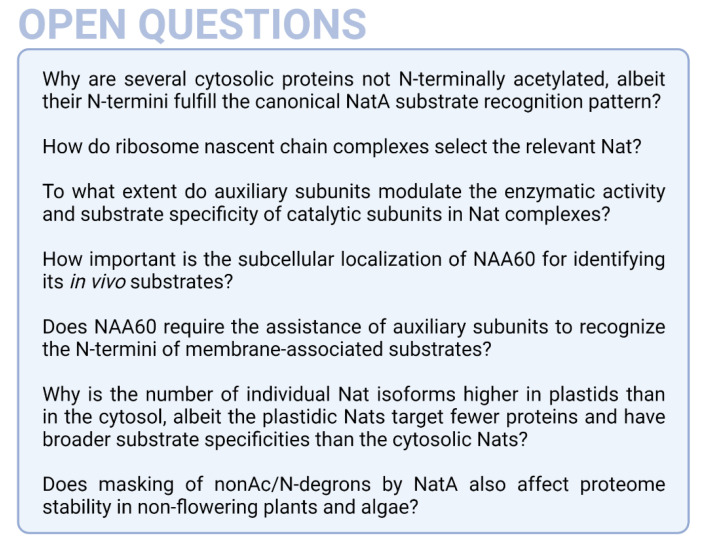
**A selection of questions aiming to understand the biological function of Nats in plants**.

So far, the research has focused on Nats tethered to ribosomes. However, many questions regarding substrate recognition at the ribosome and localization of Nats in eukaryotes remain to be unaddressed so far (see Figure 7). The ribosome-associated cytosolic Nat machinery is largely conserved among humans and plants, highlighting the importance of this co-translational modification in photosynthetic and non-photosynthetic eukaryotes. This conservation is in line with the hypothesis that eukaryotic co-translational Nats evolved from one archaeal precursor with broad substrate specificity [41]. Recent studies reveal differences between the post-translational Nat machineries of animals and plants. These differences arise at least partially from the existence of plantae-specific Nats in plastids, which evolved from cyanobacterial Nats acquired during endosymbiosis. Remarkably, these enzymes were conserved by integrating the corresponding genes into the plant nuclear genome and now function post-translationally on nuclear-encoded proteins, which are imported into the stroma [21,141]. Apart from these differences between humans and plants, there is also a clear distinction between the Nat machinery of fungi and animals. Due to divergent trajectories in the evolution of fungi and animals, only fungi underwent extensive gene loss and fission [160]. This is reflected in a reduced Nat machinery in this branch of the opisthokonts, which do not possess NAA60, and in some instances lack an enzymatically active NAA50 and the NTA facilitating protein HYPK [46,82,84].

While 20–30% of plastidic proteins are N-terminally acetylated, little is known about NTA in mitochondria [161]. Even though no Nats have been identified in this organelle so far, NTA marks were found on several mitochondrial proteins. These proteins usually localize to the outer mitochondrial membrane or intermembrane space and are co-translationally acetylated by NatC before their import into the mitochondria [132,162]. Since the import of those particular set of proteins is independent of signal peptide cleavage, the acetylation marks remain intact after the incorporation of the proteins into membranes. Considering the endosymbiotic origin of both the mitochondria and the chloroplasts, it is interesting that only cyanobacteria passed on their Nat machinery, while the GNAT-fold containing enzymes of the α-proteobacteria, which gave rise to mitochondria, apparently did not evolve into functional mitochondrial Nats. Apparently, the evolutionary pressure to preserve NTA in both organelles was different for so far unknown reasons.

Another driver of NTA diversification in different species is NatF, which is absent in fungi and localizes to different membranous compartments in humans and Arabidopsis. The C-terminal tail of the enzyme determines its localization to the Golgi apparatus in humans and the plasma membrane in plants [46,157]. While the cause of this differential localization is currently unknown, it has been speculated that *Hs*NAA60 is required to maintain the ribbon structure of the Golgi complex [14]. This ribbon structure is absent in plants allowing for the evolution of other functions of NAA60 in plants.

## 6. Future Perspectives

The depletion of the major cytosolic Nats, NatA and NatB, as well as the membrane-associated NatF results in an altered sensitivity to diverse biotic and abiotic stresses [20,48,81,121]. This suggests a role of co-translationally imprinted NTA in the rapid readjustment of the proteome to environmental cues. In this context, individual Nats have specific roles during defined stresses [20,48].

While the impact of cytosolic NTA on plant stress responses is established [81,119], the underlying molecular mechanisms remain to be identified and should be the focus of future research. Given the evidence for the influence of NTA on protein turnover, we hypothesize that altered protein stability contributes to the efficient bulk removal of stress-damaged proteins and thereby improves stress resilience (Figure 5). Alternatively, stress resilience in NatA depleted plants could be caused by affecting the stability of key regulators in the response to these stresses.

The identification of acetylation-dependent N-degrons in plants allows engineering the lifetime of individual proteins *in planta* by designing their N-terminus [74]. The application of this approach to known key stress regulators paves the road for the genetic engineering of plants with improved stress resilience, e.g., enhanced pathogen immunity by stabilization of immune receptors. Currently, this approach is superior to the genetic modification of Nats themselves since many Nats are critical for cell survival and their spatial-temporal protein interaction network at the ribosome is essential for their function but almost unknown.

## Supplementary Materials

The following supporting information can be downloaded at: https://www.mdpi.com/article/10.3390/ijms232214492/s1. Reference [163] are cited in the supplementary materials.
