# Supplementary material for: From Nucleus to Membrane: A Subcellular Map of the N-Acetylation Machinery in Plants
Source: Int J Mol Sci. 2022 Nov 21;23(22):14492. doi: 10.3390/ijms232214492 (PMC9692967; doi:10.3390/ijms232214492)
Supplement: Supplementary file 1 [file ijms-23-14492-s001.zip › ijms-2007717-supplementary.pdf]

Supplemental Figures for Pożoga et al., 2022

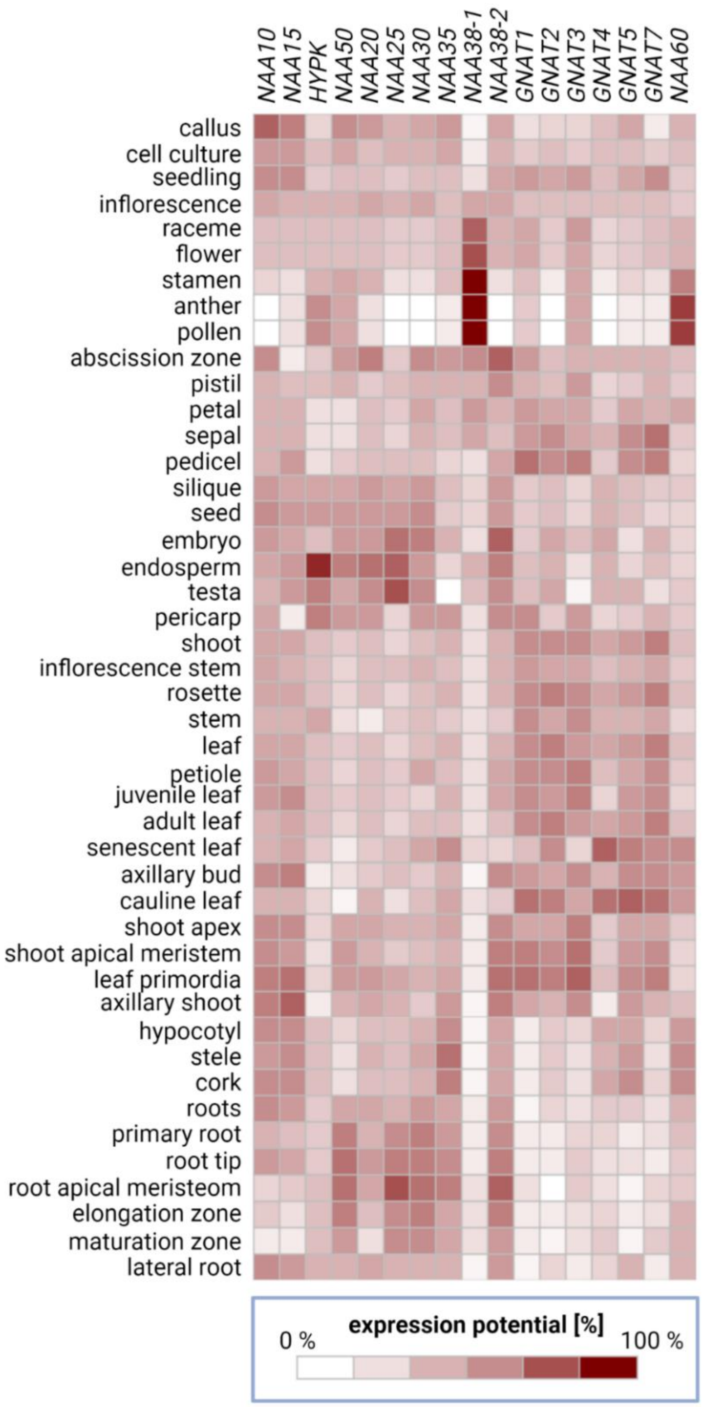

Supplemental Figure S1: Heatmap representing transcription patterns of N-acetyltransferases in different wildtype *A. thaliana* tissues. Complete set of data for wildtype Arabidopsis stored in the GENEVESTIGATOR® database was analyzed with the GENEVESTIGATOR® Anatomy tool [1].

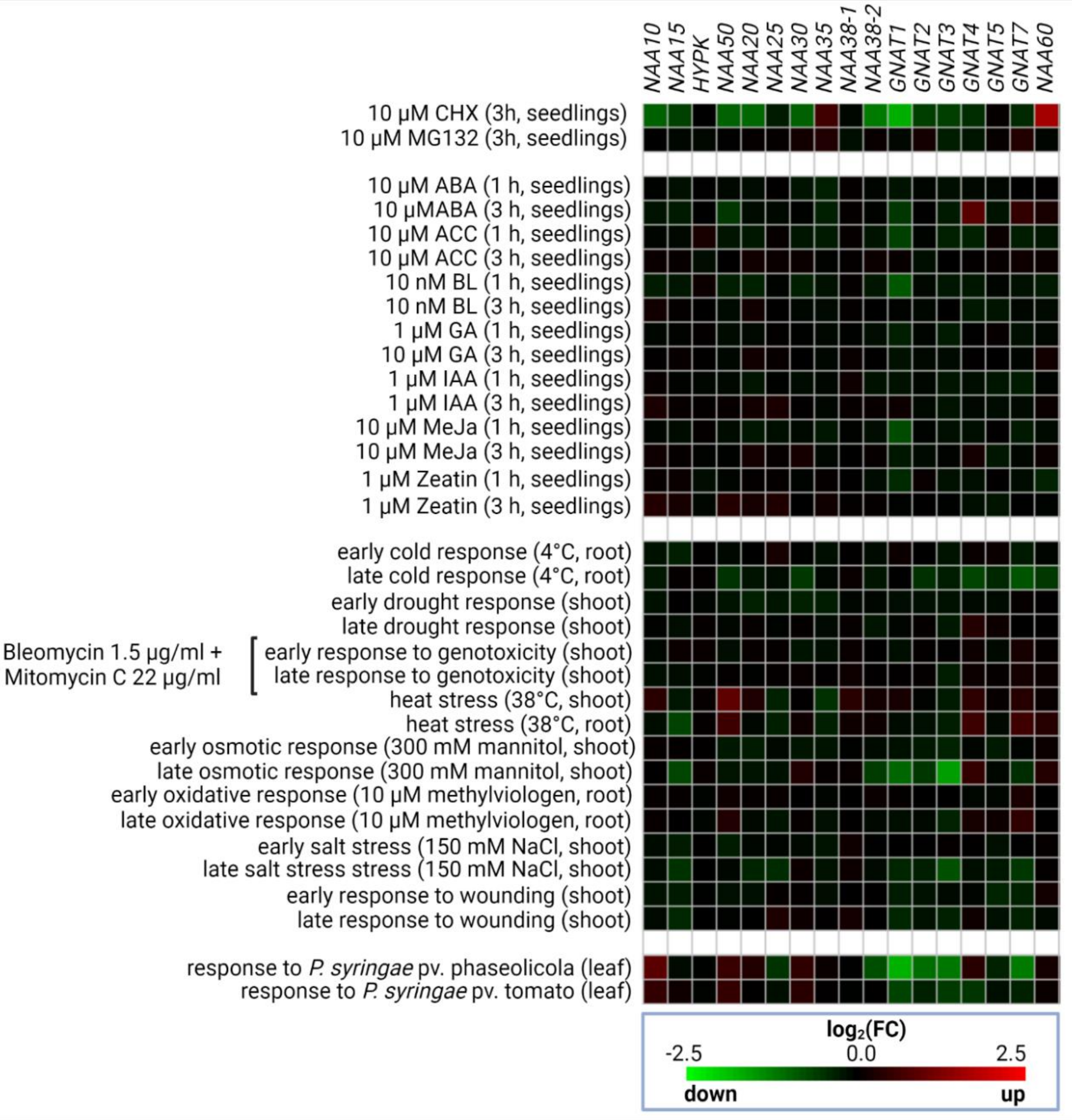

Supplemental Figure S2: Heatmap representing transcription patterns of Nat machinery components under different pharmacological treatments and stress conditions. The different datasets (experiment numbers AT-00106, AT-00110, AT-00113, AT-00120) were obtained from GENEVESTIGATOR® and analyzed with the Perturbations tool [1]. Treatments with the proteasome inhibitor MG132 and the translation inhibitor cycloheximide (CHX) were performed on seven-days-old wildtype seedlings, data and details on the experiments obtained from: TAIR:1008080804, TAIR:1008080822. The phytohormones were abbreviated as follows: ABA (abscisic acid), ACC (1-aminocyclopropane 1-carboxylic acid), BL (brassinolid), GA (gibberellic acid), IAA (indole-3-acetic acid) and MeJa (methyl jasmonic acid). Treatments were performed on seven-days-old wildtype seedlings, data and details on the experiments obtained from: TAIR:1007964750, TAIR:1007965762, TAIR:1007965859, TAIR:1007965964, TAIR:1007966040, TAIR:1007966053, TAIR:1007966175. The abiotic stress treatments were performed on 18-days-old plants. Analysis of the green tissues depicted with 'leaf', whereas analysis of root is depicted with 'root'. Data and details on the experiments obtained from: TAIR:1007966439, TAIR:1007966553, TAIR:1007966668, TAIR:1007966782, TAIR:1007966835, TAIR:1007966888, TAIR:1007966941, TAIR:1007967124. Response to bacterial pathogens was performed by leaf infiltration of five-weeks-old wildtype Arabidopsis plants. Data and details on the experiments obtained from: TAIR:1007966202.

Supplemental Reference:

1. Hruz, T.; Laule, O.; Szabo, G.; Wessendorp, F.; Bleuler, S.; Oertle, L.; Widmayer, P.; Gruissem, W.; Zimmermann, P. Genevestigator V3: a reference expression database for the meta-analysis of transcriptomes. *Adv. Bioinf.* **2008**, *2008*, 420747, doi:10.1155/2008/420747.
